# A case of pharmacist-led care team interventions to maximize rural patient quality of life

**DOI:** 10.1016/j.rcsop.2021.100004

**Published:** 2021-03-10

**Authors:** Megan Undeberg, Kimberly McKeirnan, David Easley, Kyle Frazier

**Affiliations:** a Washington State University College of Pharmacy and Pharmaceutical Sciences, United States; b CHAS Health, United States

**Keywords:** Rural patient care, Diabetes education, Patient home visit, Interprofessional care team

## Abstract

A 64-year-old rural home bound patient in Washington State was identified to be at increased risk for negative health care outcomes related to chronic end kidney disease and poorly controlled diabetes. The patient lacked understanding of the use of monitoring equipment as well as diabetes education to improve quality of health; he also did not have access to medical supply equipment. A pharmacist-led care team comprised of a pharmacist, a community health worker, and a home health nurse implemented comprehensive medication review techniques as well as direct patient care education to engage the patient in managing his health. Involvement with this home visit care team combined with the patient's recently developed interest in managing his health re-engaged the patient. He began attending more frequent visits with his providers and increased his interest in meeting with a diabetes educator at the local clinic. Resulting interactions with the patient's providers, pharmacy, and community resources increased patient's compliance, access to specialists of care, and in-home safety measures. Factors contributing to poorer overall health and higher rates of death among rural patients include increased travel time to health care facilities and providers, higher rates of unhealthy lifestyle choices such as cigarette smoking and obesity, higher rates of poverty and less access to healthcare in general. This scenario emphasizes the important role an interprofessional team plays in the care of isolated, rural health patients in managing chronic disease states for stability as well as quality of life.

## 1 Background

Residents of rural areas face numerous health disparities in comparison with those living in urban areas.^1^ People living in rural areas are more likely to die from cancer, heart disease, respiratory disease, stroke, and opioid overdoses than their urban counterparts.^1^ The U.S. Census Bureau defines a rural area as encompassing all population, housing, and territory not classified as urban (areas of 50,000 people or more).^2^ As of 2017, about 60 million Americans live in rural areas.^3^ Ninety-seven percent of the land in the United States is considered rural but only 19.3% of people live in these areas.^3^

Factors contributing to poorer overall health and higher rates of death among rural patients include increased travel time to specialty and emergency care, environmental hazards, higher rates of unhealthy lifestyle choices such as cigarette smoking and obesity, higher rates of poverty, less access to healthcare in general and lower likelihood of having health insurance.^1^^,^^4^ Socioeconomic factors also play a role. People living in rural areas tend to be poorer with an average per capita income $9242 below the average per capita income in the U.S.^5^ Approximately 25% of rural children are living in poverty and are less likely to seek care for mental health issues than children in urban areas.^1^^,^^4^^,^^5^ Rural youth are twice as likely to commit suicide.^6^

An additional contributing factor for rural patients is a lack of access to quality healthcare providers. According to the National Rural Health Association (NRHA) the patient-to-primary care physician ratio is only 39.8 to 100,000 patients in rural areas, compared to 53.3 physicians per 100,000 patients in urban areas.^5^ Access to quality care was recognized as the most frequently identified rural health priority according to Rural Healthy People 2020, the rural counterpart to Healthy People 2020.^7^ Pharmacists can help bridge the gap in rural areas and increase access to quality healthcare. In rural communities, pharmacists may be one of few healthcare providers that are regularly accessible to patients.^8^ Beyond the traditional dispensing role, pharmacists contribute to population health through the ten essential services.^9^

In Washington State ongoing work is being conducted to implement comprehensive medication review (CMR) services in rural and underserved communities.^10^ As part of a grant-funded project, rural patient CMRs were conducted by a community pharmacist at an outpatient clinic beginning Fall 2019. The project was designed for clinic physicians and nursing staff to identify and enroll up to 50 patients to be referred to a pharmacist for a CMR. Targeted patients included those who were over the age of 50 taking multiple medications and at high risk for poor health outcomes. This project was reviewed and approved by the Washington State University Institutional Review Board.

In the implementation of this project, a community pharmacist partnered with a clinic-based social worker and care coordinator. The team coordinated at-home visits with the patient, whereby the pharmacist could interview the patient regarding their actual medication use as well as visualize the physical, social, and environmental situations in which the individual patient experienced. By seeing the actual living and working environment of the patient, the pharmacist and care team were better able to determine a comprehensive care plan that moved beyond medication interventions. Safety issues were identified that were able to be resolved, as well an increased access to other social factors. It is with these team-based, home-based care visits incorporating multiple members of the healthcare team, a broad picture of the patient's capacity to accept and apply care for themselves is better formulated. When a patient comes to the clinic or the hospital, there are minimal elements of the entire patient's life available to assess. When the care is taken to the patient, a comprehensive visualization of the patient's living environment is more apparent. The objective of this work is to describe the case of a patient with poorly controlled diabetes related to a lack of education and healthcare support who was involved in this rural home health project during a six-month period.

## 2 Case presentation

The patient was referred to the pharmacy CMR service in Fall 2019 with concerns of poor management of chronic conditions especially diabetes, dizziness, and lack of resources. The care team was comprised of a pharmacist, a community health worker, and a home health nurse. This unique team is a union of integrated services from the linked community health center clinic and critical access hospital (pharmacist and nurse) plus a county-based community health worker. The community health worker typically has a pre-existing relationship with the patient, which was found to increase the level of trust with the patient and eased the acceptability of the nurse and pharmacist entering into the care of the patient in the home setting. The nurse, with clinic knowledge, and the pharmacist, with knowledge of hospital admissions, access to review the clinic medication list, and close connections with the local community pharmacy resulted in expedited, detailed reviews and interventions that would not be possible in a larger area or multi-faceted health care system seen in urban or metropolitan settings. The team of three, initially met with the patient in October 2019. The goal of the visit was to review the patient's medications and lifestyle, and to complete a CMR to identify any drug-related problems.

The patient presented as a pleasant 64-year-old Caucasian male, well known to the pharmacist from prior interactions living in a rural community. His past medical history included renal failure, benign prostatic hyperplasia (BPH) with obstruction, chronic alcohol dependance, paroxysmal atrial fibrillation, congestive heart failure (CHF), tobacco use, type 2 diabetes mellitus (T2DM), gastroesophageal reflux disease, depression, hypertension, hyperlipidemia, and chronic kidney disease (CKD). He had recently quit drinking alcohol, after many years of alcoholism but continued to smoke two packs of cigarettes daily. His main support is from his adult daughter who was living in the same community and a community health worker who took him to the grocery store regularly for food and prescriptions. Other than excursions to the grocery store, the patient was mainly home bound.

The outpatient clinic medical record had been updated during a previous clinic visit during September 2019. His medication regimen is displayed in Table 1.

**Table 1:** Patient medication regimen organized by medical condition.

Medical condition	Medication
Atrial fibrillation	Rivaroxaban 10 mg by mouth once daily
Benign prostatic hyperplasia	Tamsulosin 0.4 mg by mouth once daily
Depression	Citalopram 20 mg once daily
Diabetes mellitus type 2	Sitagliptin 100 mg by mouth once daily
	Liraglutide pen 1.2 mg injected SQ once daily
Edema and supplementation for potassium excretion	Furosemide 40 mg by mouth once daily
	Potassium chloride 20 mEq, two tablets (40 mEq) twice daily
Gastroesophageal reflux disease	Omeprazole 20 mg by mouth once daily
Hyperlipidemia	Atorvastatin 40 mg by mouth once daily
	Fenofibrate 160 my by mouth once daily
Hypertension and heart failure	Spironolactone 50 mg by mouth once daily
	Carvedilol 25 mg by mouth twice daily
Insomnia	Zolpidem 10 mg once daily in the evening as needed
Vitamin deficiency	Folic acid 1 mg by mouth once daily

### 2.1 Pharmacist intervention

The first home visit occurred in October 2019. The patient was pleased to have the care team come to his home and expressed a recent increase in motivation to improve his blood glucose, eat healthier food, and work on taking better care of himself. Notably, when the pharmacist, community health worker, and nurse entered the patient's home they observed that his bedding was on the floor. He reported that he did not have a bed and had been sleeping on the floor. When the pharmacist asked about this the patient reported experiencing dizziness when getting up from his bedding on the floor. The pharmacist immediately had concerns that the patient was experiencing orthostatic hypotension which was especially problematic since he was getting up off the floor rather than getting out of a bed.

The care team interviewed the patient about the types of food the patient regularly ate, as well as options such as fruits and vegetables. The patient was pleased to report he had been “eating a lot more fruit” recently; when asked what kind of fruit, he permitted the team to open his refrigerator to see. The care team found that patient was correctly reporting that he had been eating fruit, but it was the canned variety in extra heavy syrup. The nurse and pharmacist provided education to the patient about the amount of sugar present in canned fruit with heavy syrup and recommended alternative options for the patient that had less sugar.

Continuing through the review, the pharmacist inquired to the pattern of blood glucose monitoring by the patient. The patient replied that he had monitored his blood glucose in the past but had a been given new meter during his clinic visit last month. The blood glucose meter looked brand new and the patient was still storing it in the original packaging. Upon further discussion, it became evident that the patient was never instructed on how to use it the blood glucose meter and did not know how to turn it on. The pharmacist assisted the patient in turning on the new blood glucose meter and walked through the operation of the machine with him. They tested his blood glucose together and found it to be elevated at 270 mg/dL.

During the CMR visit, the pharmacist found the majority of the medications reported in the medical record were present and available, but the patient could not articulate a consistent regimen for how he was taking his medication. The pharmacist was immediately concerned with the patient's ability to manage his medication. The pharmacist reviewed the patient's medication box to confirm contents and provided education about the role of each prescription with him. The zolpidem, spironolactone, and sitagliptin were missing from the medications at the patient's home and the patient was not aware of whether he was supposed to be taking these medications.

### 2.2 Outcome

After the home visit was completed, the pharmacist reviewed the patient's medication in the clinic electronic health record (EHR) to determine why the patient was reported to be taking zolpidem, spironolactone, and sitagliptin but he did not have these medications in his home. The EHR confirmed these medications were listed but when the pharmacists called the local community pharmacy to confirm the medication list, the community pharmacist did not have any record of filling these medications. Additionally, it was found that the community pharmacy had been dispensing rivaroxaban 15 mg rather than the prescribed 10 mg dosage. The results of the home visit and medication review were discussed with the patient's primary care physician. The medication changes made by the pharmacist with approval from the primary care physician are displayed in Table 2.

**Table 2:** Medication changes made by pharmacist organized by medical issue.

Medical issue	Medication change made by pharmacist
Orthostatic hypotension and fall risk	• Discontinuation of tamsulosin • Therapeutic interchange of mirtazapine 15 mg by mouth once daily in the evening to replace zolpidem
Dispensing error	• Clarification of rivaroxaban dose of 10 mg by mouth once daily
Poorly controlled diabetes	• Initiation of canagliflozin 100 mg by mouth once daily in the morning • Initiation of insulin glargine 18 units injected subcutaneously once daily in the evening

Additional interventions made by the pharmacist included setting up weekly pill packs from the local community pharmacy with a four-times-a-day medication regimen and procuring a hospital bed to assist the patient in minimization of orthostasis, prevention of fall risk, and improvement in safety. The pharmacist knew of a hospital bed that was previously owned by another community member who was now deceased. The pharmacist was able to obtain the bed for this patient, but it took several weeks to coordinate a team of people to move the bed, which was very heavy.

Two months later, in December 2019, the pharmacist returned for a second home visit. At this follow up visit, the pharmacist confirmed that the medication changes from the previous visit had been implemented and ensured the patient had the medication pill packs from the local pharmacy. The patient now had consistent refill patterns with all medications. The patient had the pill packs and appeared to be taking his medication appropriately, but he could not articulate the role or use of the individual medications. The pharmacist used this opportunity to discuss each medication with him.

Involvement in this care team home visit project combined with his recently developed interest in managing his health re-engaged the patient in attending more frequent visits with his providers and increased his interest in meeting with a diabetes educator at the local clinic. Despite the increased awareness and engagement of the patient, his kidneys continued to fail. After the December visit with the pharmacist, it was determined the patient was in kidney failure. He decided not to pursue hemodialysis and opted to enroll in palliative care for end-stage renal disease in December 2019.

In February 2020, the patient met with the diabetes educator at the clinic. His blood glucose was 150 mg/dL at that visit and leading up to that the ranges were in the 110 to 120 mg/dL. Home health nurses maintained contact weekly via phone to check in with the patient and provide support. His home health manager continued to see him and take him shopping for groceries. The patient continued to have stability and adherence with his medication regimen.

Even though his kidney disease continued to advance, in early 2020 the patient felt well enough to resume one of his favorite pastimes: playing his guitar in the evenings at the local bar. This he continued until the COVID-19 restrictions were put in place by the governor of the state in March 2020. Ultimately, the patient died from complications of his medical conditions in early summer 2020.

## 3 Discussion

The patient was referred for a care team home visit and presented as a non-compliant patient with uncontrolled diabetes and orthostatic hypotension. Upon meeting with the patient, it was discovered he was engaged and willing to make lifestyle and management changes to improve his health, even at late, advanced stage of his chronic diseases. The patient had been given the appropriate tools for management of his diabetes but had been left uneducated on how to utilize the blood glucose meter or how to make appropriate dietary choices. He also lacked the resources to minimize fall risk and was sleeping on the floor.

With engagement of the care team, and the pharmacist review, the patient received vital education. He learned how to use his blood glucose meter to monitor his diabetes, how to make wise food choices to support his diabetes and kidney health, gained understanding in the role of each of his medications, and obtained an updated medication regimen that minimized orthostatic hypotension as well as intensified his diabetes regimen to improve his quality of life. The pharmacist also set-in motion a series of conversations within the community to access a hospital bed for use by the patient.

Healthy People 2020 strives for “health equity and elimination of disparities” as well as creation of environments that support healthy lifestyles plus promotion of quality of life across all age groups and stages.^11^ In rural areas such as described here, providers and patients frequently struggle with access to health care systems, related to simple factors such as weather and transportation up to more complicated factors such as underlying mental and physical health issues that prevent the patient from leaving their home. Bringing the system to them bridges this gap; bringing the pharmacist to the home health visit adds an integrative level of care that can further identify risk factors for a patient managing multiple chronic medical conditions at home.

Comprehensive medication reviews and their value-added service has been documented in a variety of care settings, not only in the United States, but also throughout varied settings throughout the world.^12^, ^13^, ^14^, ^15^ Novel to this project is the creation of the team and implementation of the service in a rural area. The difference here is encouraging the face-to-face team visit incorporating a pharmacist-led comprehensive medication review along with a visiting nurse visit and social community care worker check-in. However, the ultimate goal of comprehensive medication reviews, regardless of the patient setting is to provide a purposeful review of the individual's medication regimen, and work directly with the patient and their care team to maintain or improve the overall health and wellbeing of the patient. This patient met with the care team during a 5-month period between October 2019 and the state-mandated COVID-19 shutdown in March 2020. Not only was the patient stabilized in his later stages of diseases, but he also experienced the ability to resume “normal” community-based activities that he previously was unable to enjoy due to limitations by his disease state ramifications.

This work was possible through a grant-funded project designed to increase rural patient access to quality healthcare.^10^ Because this work was funded, the project focus was on demonstrating the benefit of the pharmacist as part of a home visit healthcare team rather than on cost savings or financial benefits. Identifying ways to bill for these services to make this work sustainable beyond the funded project is an ongoing effort. Additionally, results of this work may not be generalizable in urban areas where providers and resources are more easily accessible.

The project pharmacist reflected on his relationship with this patient and was disappointed that he had not been asked to meet with the patient earlier in his disease progression. Even substantial interventions by the healthcare team during the fall of 2019 were not enough to save the life of this patient whose diseases had already progressed toward end-stage. By creating more opportunities for pharmacists to engage rural patients in home visits at an earlier stage, there is potential to improve patient outcomes rather than just improving quality at the end of life.

## 4 Conclusion

This case demonstrates the vital role of the care team that incorporates pharmacists with other health care providers to support the care of medically complex rural patients. With the interventions of the pharmacist, and weekly support of the care team, the patient experienced improved quality of life and resumption of activities that interested him, even as the patient progressed through end-stage renal disease.

## Funding

This project was funded in part by the Empire Health Foundation, Spokane, Washington, USA. The content is solely the work of the authors and does not necessarily represent the views of the Empire Health Foundation.

## Declaration of Competing Interest

The authors do not have any conflicts of interest or financial interests to disclose.
